# Upregulation of the Outer Membrane Protein OmpV and Its Role in Polymyxin B Stress Adaptation in *Vibrio mimicus*

**DOI:** 10.3390/microorganisms14071527

**Published:** 2026-07-13

**Authors:** Shin-ichi Miyoshi, Yuta Onodera, Shunki Annoi, Basilua Andre Muzembo, Daisuke Imamura

**Affiliations:** 1 Graduate School of Medicine, Dentistry and Pharmaceutical Sciences, Okayama University, 1-1-1, Tsushima-Naka, Kita-Ku, Okayama 700-8530, Okayama, Japan; 2 Research Center for Intestinal Health Science, Okayama University, 1-1-1, Tsushima-Naka, Kita-Ku, Okayama 700-8530, Okayama, Japan; 3 Research Institute of Nursing Care for People and Community, University of Hyogo, 13-71 Kitaoji-Cho, Akashi 673-8588, Hyogo, Japan

**Keywords:** *Vibrio mimicus*, polymyxin B, OmpV, β-barrel protein, antibiotic sequestration

## Abstract

OmpV is an outer membrane protein found in a variety of Gram-negative bacteria. In this study, we demonstrated that *V. mimicus* strain CS-66, isolated from a patient with diarrhea, was resistant to polymyxin B (PL-B) and colistin (CL) but susceptible to chloramphenicol and ciprofloxacin. When strain CS-66 was cultured in Luria–Bertani broth containing a sub-minimal inhibitory concentration of each antibiotic used for the susceptibility tests, *ompV* expression as assayed by reverse transcription-qPCR, significantly increased regardless of the antibiotic tested. In contrast, cell aggregation during the early log phase was observed only when the strain was grown in the broth supplemented with PL-B or CL. A space-filling model of *V. mimicus* OmpV was generated. The model showed that, although OmpV adopted a β-barrel conformation, it had closed top and bottom ends and possessed a lateral cavity. Structural analysis further indicated that the interior of this lateral cavity was negatively charged and sufficiently large to accommodate PL-B. These results suggest that *V. mimicus* may adapt to PL-B stress through physical sequestration within the negatively charged cavity of OmpV.

## 1. Introduction

Outer membrane proteins (OMPs) of Gram-negative bacteria perform multiple functions, including regulating the transport of nutrients and minerals, preventing the entry of antibiotics and toxins, sensing and transmitting environmental signals, and facilitating bacterial adhesion to host surfaces [1,2]. OmpV is one of the most common OMPs, with a molecular weight of 26 kDa [3,4]. Although its fundamental functions remain unclear, OmpV has been reported to respond to changes in environmental osmotic pressure in *Photobacterium damselae* [5], *Vibrio parahaemolyticus* [6] and *Vibrio alginolyticus* [7]. For instance, OmpV production in *V. parahaemolyticus* was significantly higher at 0.85% NaCl than at 3.5% NaCl [6]. In addition, OmpV has been suggested to be associated with kanamycin (KM) resistance in *Escherichia coli* [8] and to facilitate bacterial attachment to human intestinal epithelial cells in *Salmonella* Typhimurium [4,9].

Our previous study [10] demonstrated that OmpV production was upregulated in *Vibrio mimicus* (a pathogen that causes diarrhea in humans [11]), when the bacterium was exposed to a low concentration of polymyxin B (PL-B). This finding suggests that OmpV contributes to bacterial adaptation to PL-B stress. PL-B is a cyclic antimicrobial peptide containing both cationic and hydrophobic amino acid residues, which exerts bactericidal activity by binding tightly to the negatively charged bacterial cell surface and ultimately disrupting the cell membrane through the formation of transmembrane pores [12]. *Vibrio cholerae* (a causative agent of cholera [11]), is a species closely related to *V. mimicus* [13]. This pathogen possesses the *carRS* (*vprAB*) operon upstream of the *ompV* gene [14,15]. The CarR and CarS proteins form a two-component regulatory system that has been reported to be associated with PL-B resistance [14,15]. Specifically, the CarRS system enhances the expression of the *almEFG* operon, resulting in the glycylation of lipopolysaccharide (LPS) in the outer membrane via the AlmEFG pathway. Glycylation reduces the negative charge of LPS and inhibits the binding of PL-B to the outer membrane. Similarly to *V. cholerae*, the *carRS* operon is located upstream of *ompV* in the chromosome of *V. mimicus*; however, it remains unclear whether CarRS is involved in PL-B resistance in this species.

In this study, we examined whether OmpV contributes to the adaptation of *V. mimicus* to PL-B stress. We demonstrate that *ompV* expression is significantly increased, whereas *carRS* expression remains unchanged, when *V. mimicus* is cultured in the presence of PL-B at a sub-minimum inhibitory concentration (sub-MIC). A space-filling model of *V. mimicus* OmpV reveals that it has closed top and bottom ends and possesses a lateral cavity. Additional structural analyses indicate that the interior of this lateral cavity is negatively charged and sufficiently large to accommodate PL-B.

## 2. Materials and Methods

### 2.1. Antibiotics and Antimicrobial Agents

In the present study, the following antibiotics were used: PL-B (Fujifilm Wako Pure Chemical, Osaka, Japan), colistin (CL) (Fujifilm Wako Pure Chemical), kanamycin (KM) (Tokyo Chemical Industry, Tokyo, Japan), chloramphenicol (CP) (Nacalai Tesque, Kyoto, Japan), and ciprofloxacin (CPFX) (ICN Biomedicals, Aurora, OH, USA). Additionally, deoxycholic acid (DCA) (Fujifilm Wako Pure Chemical), human LL-37 (Peptide Institute, Ibaraki, Japan), and α-defensin-5 (HD-5) (Peptide Institute) were used.

### 2.2. Determination of MICs and Sub-MICs

The MIC of each antibiotic was determined by the broth microdilution method according to the standard protocol of the Japanese Society of Chemotherapy [16]. *V. mimicus* strain CS-66, isolated from a patient with diarrhea in New Orleans, LA, USA, was cultured in 5 mL of Luria–Bertani (LB) broth (1.0% tryptone [Thermo Fisher Scientific, Waltham, MA, USA], 0.5% yeast extract [Thermo Fisher Scientific], and 0.5% NaCl; pH 7.5) until the mid-log phase (OD600 = 0.5). After cultivation, the bacterial suspension was adjusted to a turbidity equivalent to a 0.5 McFarland standard (0.5 MFU) and subsequently diluted 10-fold. An aliquot (10 μL) of the prepared bacterial suspension was added to each well of a 96-well plate containing 0.2 mL of LB broth supplemented with an antibiotic at concentrations ranging from 0 to 100 μg/mL. The plate was incubated statically at 37 °C for 24 h, and bacterial growth (OD655) was measured with an iMark microplate reader (Bio Rad, Hercules, CA, USA). Thereafter, the MIC and sub-MIC (approximately one-third of the MIC) of each antibiotic were determined. Based on the determined MIC values, the resistance level of strain CS-66 to each antibiotic was classified according to the CLSI M100 (34th Edition) guidelines [17].

We also examined the MICs of non-antibiotic substances. An aliquot (10 μL) of the prepared bacterial suspension was added to each well of a 96-well plate containing 0.2 mL of LB broth supplemented with 0–100 μg/mL of a human intestinal factor (LL-37, HD-5, or DCA) or 0–9% NaCl. The plate was incubated statically at 37 °C for 24 h, and the bacterial growth was measured.

### 2.3. Reverse Transcription-qPCR (RT-qPCR)

Strain CS-66 was cultured in LB broth supplemented with a sub-MIC of each antibiotic until the mid-log phase. An aliquot (0.5 mL) of the bacterial culture was mixed with 1.0 mL of RNAprotect Bacteria Reagent (Qiagen, Venlo, The Netherlands) and incubated at room temperature for 10 min. The sample was centrifuged at 5000× *g* for 10 min, and the bacterial cells were collected and suspended in 0.2 mL of TE buffer (10 mM Tris-HCl, 1 mM EDTA, pH 8.0) containing lysozyme (15 mg/mL), followed by incubation at room temperature for 10 min. Total RNA was subsequently extracted using the RNeasy Mini Kit (Qiagen) according to the manufacturer’s instructions.

For the RT reaction to synthesize cDNA [18], 4.0 μL of the RNA sample (50 ng/μL) was mixed with 4.0 μL of LunaScript RT SuperMix (New England Biolabs, Ipswich, MA, USA) and 12 μL of nuclease-free water. The resulting reaction mixture (20 μL) was then placed in a GeneAtlas482 thermal cycler (Astec, Fukuoka, Japan) and subjected to the reaction with the following conditions: primer annealing at 25 °C for 2 min, cDNA synthesis at 55 °C for 10 min, and heat inactivation at 95 °C for 1 min.

Appropriate primer sets for amplifying the specific target cDNAs (Table 1) were designed based on the genome sequence of *V. mimicus* strain NCTC11435 (GenBank accession number: GCA_900460385/NZ_UHIG00000000). For qPCR analysis [18], 1.0 μL of the cDNA sample was mixed with 10 μL of Luna Universal qPCR Master Mix (New England Biolabs), 1.0 μL (0.5 μL each) of the primer set (10 μM), and 8.0 μL of nuclease-free water. The prepared qPCR mixture (20 μL) was loaded onto a Thermal Cycler Dice Real Time System Lite (Takara Bio, Kusatsu, Japan) and subjected to an initial denaturation at 95 °C for 1 min, followed by 40 cycles of denaturation at 95 °C for 15 sec and annealing/extension at 60 °C for 30 sec. Thereafter, the amount of target cDNA was quantified based on the threshold cycle (Ct) value. In all qPCR experiments, the gene encoding malate dehydrogenase (*mdh*), a housekeeping gene, was used as an internal control [10].

### 2.4. Structural Analysis of OmpV

The *V. mimicus* OmpV consists of 257 amino acid residues, of which the N-terminal 19 residues were predicted to constitute a signal peptide. Therefore, the remaining 238 residues were used to construct the tertiary structure using AlphaFold 3 [19]. Subsequently, a space-filling model of OmpV was generated using UCSF ChimeraX [20]. Additionally, the surface electrostatic potential and cavity volume were analyzed using Adaptive Poisson-Boltzmann Solver (APBS) [21] and CASTpFold [22], respectively.

## 3. Results and Discussion

### 3.1. MICs of Antibiotics for V. mimicus Strain CS-66

Each antibiotic was serially 2-fold diluted in the wells of a microtiter plate containing LB broth, and a cell suspension of *V. mimicus* strain CS-66 was added to each well. After static cultivation at 37 °C for 24 h, bacterial growth was assessed to determine the MICs (Table 2). The MICs for PL-B and CL, which share a similar mechanism of action, were both 6.25 μg/mL. In contrast, the MICs of CP and CPFX were lower (1.56 μg/mL and 0.00625 μg/mL, respectively). The antibiotic resistance profile of strain CS-66 was determined based on the obtained MICs and the breakpoints for *Enterobacterales* published in CLSI M100 [17]. As shown in Table 2, *V. mimicus* strain CS-66 was susceptible to CP and CPFX but resistant to PL-B and CL. Sub-MICs (approximately one-third of the MICs) of the antibiotics were also calculated for subsequent experiments (Table 2).

We also examined the MICs of non-antibiotic substances. The human intestinal factors (LL-37, HD-5 and DCA) exhibited no antibacterial activity, even at concentrations up to 100 μg/mL. However, NaCl markedly inhibited the growth of *V. mimicus* at a concentration of 6.0%.

### 3.2. Expression of ompV and Its Neighboring Genes

**Figure 1:**
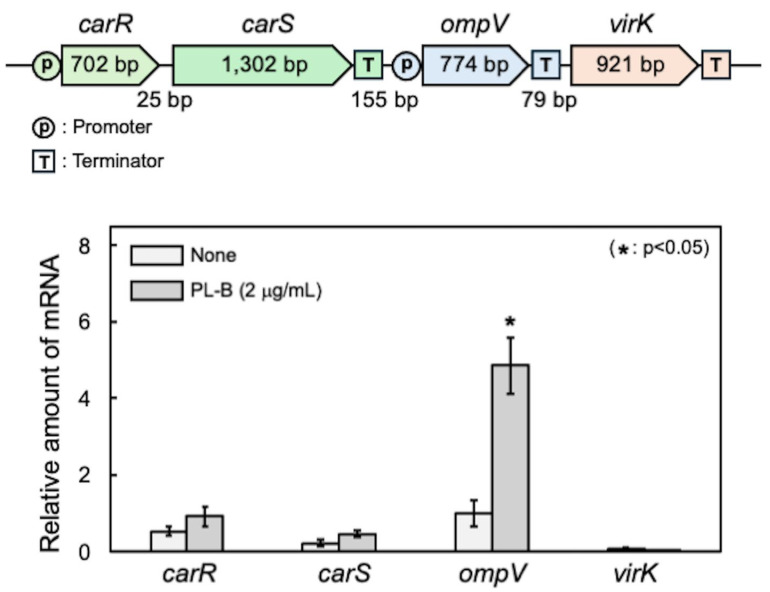
**Expression of *ompV* and its neighboring genes in *V. mimicus* strain CS-66 exposed to a sub-MIC of PL-B.** Strain CS-66 was cultured in LB broth supplemented with a sub-MIC of PL-B. Total RNA was prepared at the mid-log phase, and the expression levels (mRNA levels) of *ompV* and its neighboring genes were quantified by RT-qPCR (*n* = 8). The expression level of *ompV* in the absence of PL-B was set to 1.0.

We previously documented that OmpV production increased following exposure to a sub-MIC of PL-B [10]. To clarify whether this increase was attributable to enhanced gene expression, RT-qPCR was performed using RNA extracted from strain CS-66 cells exposed to a sub-MIC (2.0 μg/mL) of PL-B. The results showed that *ompV* expression increased in 4.8-fold when the cells were cultured in the presence of PL-B (Figure 1).

As in *V. cholerae* [14,15], the *carRS* operon is located upstream of *ompV* on the *V. mimicus* chromosome (Figure 1). Because the CarRS regulatory system is known to be involved in PL-B resistance in *V. cholerae* [14,15] and *Vibrio vulnificus* [23], we measured the expression levels of *carRS* along with *virK*, which is located downstream of *ompV* (Figure 1). In the presence of a sub-MIC of PL-B, *carRS* expression was slightly elevated; however, the difference was not statistically significant. This finding suggests that PL-B resistance in *V. mimicus* does not mainly depend on the CarRS system. In *V. cholerae*, the *virK* gene forms an operon with *carRS* and *ompV* [24]. In contrast, the *V. mimicus virK* gene did not form an operon with these genes (Figure 1). Additionally, due to the absence of a putative promoter sequence upstream of the *virK* gene, its expression was negligible even in the presence of PL-B (Figure 1).

Therefore, in *V. mimicus*, the *ompV* gene is transcribed as a single unit, and OmpV may function independently of the CarRS system. OmpV is a common outer membrane protein among various *Vibrio* species. However, analyses using the NCBI databases, including the Basic Local Alignment Search Tool [25], indicated that only four species (*V. cholerae*, *Vibrio metoecus*, *V. mimicus*, and *Vibrio paracholerae*) possess the *carRS*-*ompV*-*virK* gene cluster. This finding supports our conclusion that OmpV is not functionally associated with CarR, CarS or VirK, even though these genes form an operon in *V. cholerae*.

### 3.3. Effects of Antibiotics and Non-Antibiotics on the Expression of ompV

**Figure 2:**
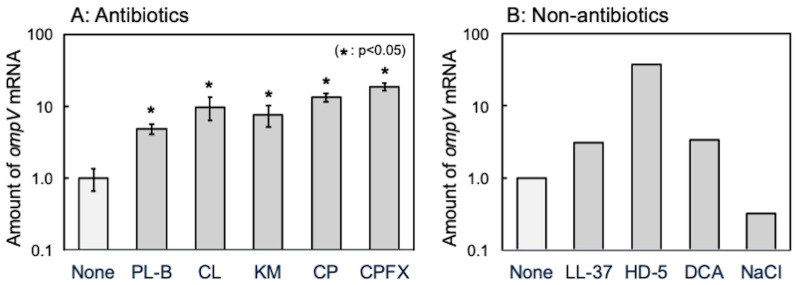
**Expression of *ompV* in *V. mimicus* strain CS-66 exposed to sub-MICs of antibiotics or non-antibiotic substances**. Strain CS-66 was cultured in LB broth supplemented with a sub-MIC of an antibiotic or a non-antibiotic substance. Total RNA was prepared at the mid-log phase, and *ompV* expression levels were quantified by RT-qPCR. The RT-qPCR assays were performed in duplicate. The experiments using antibiotics (**A**), and non-antibiotic substances (**B**) were repeated three and two times, respectively.

Strain CS-66 was cultured in LB broth supplemented with a sub-MIC of each antibiotic, and total RNA was extracted at the mid-log phase to quantify *ompV* mRNA expression by RT-qPCR. The *ompV* expression level increased 5-fold (PL-B), 10-fold (CL), 8-fold (KM), 13-fold (CP), and 18-fold (CPFX) (Figure 2A). Thus, *ompV* expression was consistently upregulated regardless of bacterial susceptibility to the antibiotics tested (*p* < 0.05).

We also tested the effects of several non-antibiotic substances on *ompV* expression (Figure 2B). Two human intestinal antimicrobial peptides (LL-37 and HD-5; 100 μg/mL) and an intestinal bactericidal substance (DCA; 100 μg/mL) similarly increased the *ompV* expression. In contrast, as reported for other bacterial species, including *Vibrio* species [5,6,7], NaCl (2.0%) reduced *ompV* expression in *V. mimicus*.

These results indicate that *V. mimicus* may regulate *ompV* expression in response to various environmental stresses. Consequently, OmpV in *V. mimicus* may function as an outer membrane sensor that detects various extracellular antibiotics and intestinal factors, thereby regulating cellular metabolic processes to mitigate their detrimental effects.

### 3.4. Aggregation of Bacterial Cells During the Early Growth Phase

**Figure 3:**
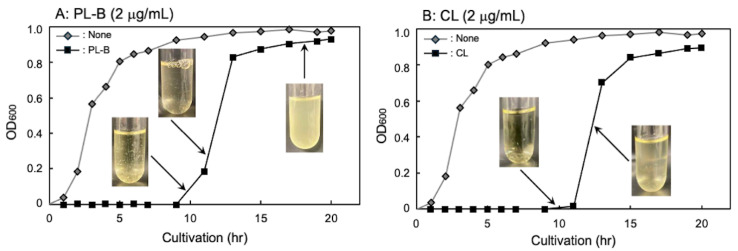
**Growth and cell aggregation of *V. mimicus* strain CS-66.** Strain CS-66 was cultured in LB broth supplemented with sub-MICs of PL-B (**A**) or CL (**B**), and the growth (OD600) was measured periodically (*n* = 2).

**Figure 4:**
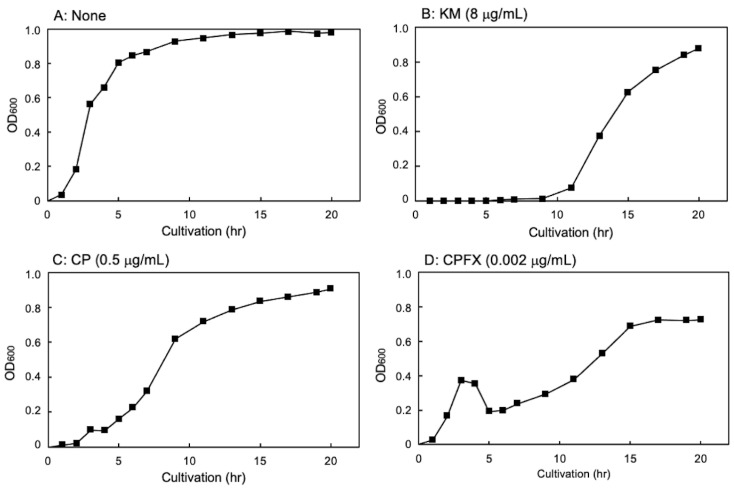
**Growth of *V. mimicus* strain CS-66 in the presence of antibiotics.** Strain CS-66 was cultured in LB broth supplemented with sub-MICs of KM, CP, or CPFX. The growth (OD600) was measured periodically (*n* = 2).

The growth curves of strain CS-66 in the presence or absence of sub-MICs of antibiotics are shown in Figure 3 and Figure 4. When strain CS-66 was inoculated into LB broth, bacterial multiplication was observed within 1 h, and growth reached the mid-log phase after 3 h of cultivation (Table 3). In the broth supplemented with sub-MICs of PL-B, CL, or KM, bacterial growth was markedly suppressed; specifically, the lag phase was prolonged to 9 h, and growth reached the mid-log phase after 11–15 h of cultivation (Table 3). However, neither PL-B nor CL affected the generation time of strain CS-66 (Table 3). In contrast, in the broth containing CP or CPFX, bacterial generation time was significantly prolonged, although the duration of the lag phase remained unchanged (Table 3).

In the bacterial growth experiments, cell aggregation was observed during the early log phase in the broth containing either PL-B (Figure 3A) or CL (Figure 3B). However, after the mid-log phase, the cultures became uniformly turbid due to the marked proliferation of strain CS-66. In contrast, no cell aggregation was observed in cultures exposed to sub-MICs of KM, CP, or CPFX. These results suggest that PL-B and CL, both of which are cationic peptides, associate directly with negatively charged substances on the bacterial cell surface, thereby either neutralizing the cell surface charge and increasing bacterial cell hydrophobicity, or alternatively triggering the bacterial stress response via a distinct pathway. Additional microscopic analysis is also required to clarify whether morphological changes in the cells are induced by exposure to sub-MICs of PL-B or CL.

Aggregate formation can cause a local increase in bacterial cell density and may lead to the development of biofilms. Therefore, cell aggregation may represent a survival strategy of *V. mimicus* strain CS-66 against antibiotic attack by shielding the bacterial population from direct contact with antibiotics. Similar phenomena have been reported in *Pseudomonas aeruginosa* [26] and *Staphylococcus aureus* [27], in which cell aggregation conferred resistance to various antibiotics. To clarify the relationship between cell aggregation and antibiotic resistance, for instance, it will be necessary to form antibody-mediated cell aggregates and compare their MICs against various antibiotics with those of planktonic cells of strain CS-66.

### 3.5. Structural Analyses of OmpV

**Figure 5:**
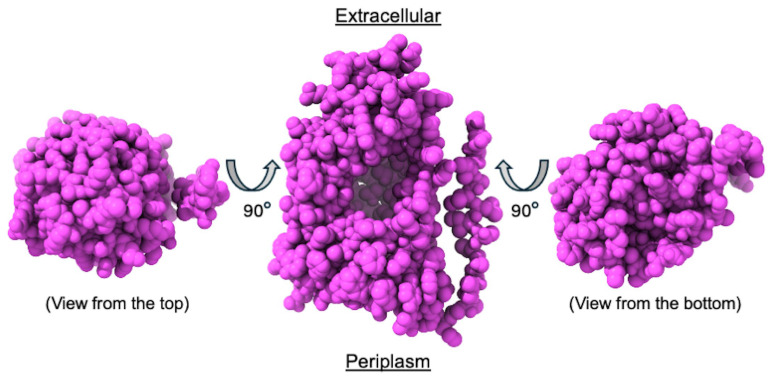
**Space-filling model of *V. mimicus* OmpV.** A tertiary structure of OmpV was predicted using AlphaFold 3, and a space-filling model was generated using UCSF ChimeraX. The left panel shows the top view, and the right model shows the bottom view.

We predicted the tertiary structure of *V. mimicus* OmpV using AlphaFold 3. The prediction was performed on a mature polypeptide consisting of 238 amino acid residues, generated by removing the predicted N-terminal signal peptide from the full-length sequence of 257 residues. A space-filling model was then generated using UCSF ChimeraX (Figure 5). The model revealed that, although *V. mimicus* OmpV adopted a β-barrel structure, it possessed a cavity with a wide lateral opening. Recently, Mathieu-Denoncourt et al. [24] reported that OmpV from *V. cholerae* contains a similar cavity. Interestingly, both the top and bottom ends of *V. mimicus* OmpV appeared to be closed. Therefore, unlike typical OMPs acting as porins, *V. mimicus* OmpV may not form a hollow tubular structure. However, we cannot rule out the possibility that the association of a small molecule with the cavity induces a structural change in *V. mimicus* OmpV, which allows it to function as an efflux pump or a porin.

**Figure 6:**
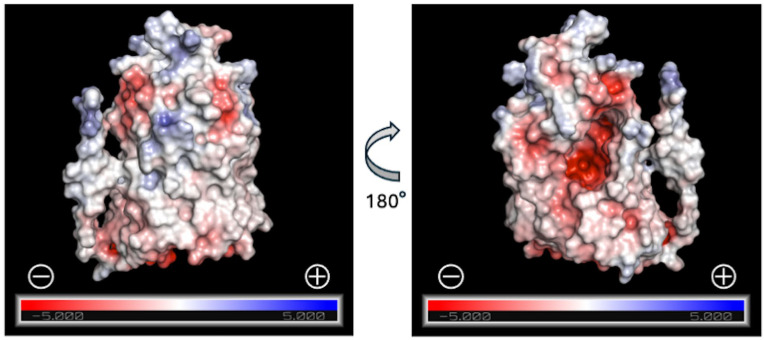
**Surface electrostatic potential of *V. mimicus* OmpV.** The surface of OmpV was colored based on the electrostatic potential calculated by APBS analysis, ranging from −5 kT/e (red) to 5 kT/e (blue).

As shown in Figure 6, electrostatic potential analysis using APBS indicated that the interior of the cavity was negatively charged, suggesting that it may serve as a potential binding site for small cationic compounds such as PL-B. In addition, analysis using CASTpFold revealed that the major and minor axes of the cavity opening of *V. mimicus* OmpV were 20 Å and 10 Å, respectively. These dimensions exceed those of PL-B (17 Å and 9 Å). Furthermore, the cavity volume was estimated to be 1800 Å^3^, which is sufficiently large to capture a PL-B molecule (volume: 1050 Å^3^).

Taken together, these findings suggest that *V. mimicus* may adapt to PL-B stress through the physical sequestration of the antibiotic within the OmpV lateral cavity, which has a negatively charged interior. However, to further validate these findings, molecular docking simulations of PL-B into the OmpV cavity will be required. Additionally, to confirm the physical interaction between isolated OmpV and PL-B, experiments using isothermal titration calorimetry or surface plasmon resonance will be necessary.

Although the *ompV* gene is conserved across many species in the genus *Vibrio*, the functional roles of the OmpV protein may vary among individual species. For instance, *V*. *vulnificus* exhibits a remarkably high intrinsic resistance to PL-B, with a MIC of 64 μg/mL [10], which is 10 times higher than that of *V. mimicus* (Table 2). Nevertheless, incubation with a sub-MIC of PL-B fails to induce OmpV production [10].

## 4. Conclusions

*V. mimicus* strain CS-66 was resistant to PL-B and CL but susceptible to CP and CPFX. Cultivation in the presence of these antibiotics significantly increased *ompV* expression, regardless of the bacterial susceptibility to the tested antibiotics. However, cell aggregation during the early growth phase was observed only in the presence of PL-B or CL. Structural modeling predicted that, although OmpV in *V. mimicus* adopted a β-barrel structure, it possessed a lateral cavity and closed extracellular and periplasmic ends. Furthermore, the interior of the cavity was predicted to be negatively charged, and the cavity was sufficiently large to accommodate PL-B. Taken together, these results suggest that *V. mimicus* acquires PL-B adaptation by physically sequestering the antibiotic within the cavity of OmpV. Although bacterial OMPs are widely known to function as efflux pumps or porins due to their hollow tubular structures, the results of this study suggest the existence of non-hollow structured OMPs possessing a lateral cavity, which may sequester small molecules.

## Figures and Tables

**Table 1:** Oligonucleotide primers used for qPCR.

Gene	Locus *	Primer	Nucleotide Sequence	Position
*ompV*	SUP10251	Sense	5′-CACCTACTTGACTGGCAGCG-3′	273-292
		Antisense	5′-AAGAGCGATATCGGCGTTCA-3′	368-387
*carR*	SUP10255	Sense	5′-GATTTTGACCACGTGGCTGC-3′	259-278
		Antisense	5′-TTACTCGTTCTTCTCGGCGC-3′	357-376
*carS*	SUP10253	Sense	5′-GTGGCAAGCTGAAGTGTTGG-3′	696-715
		Antisense	5′-ACAGCTCATCGACCATGGTC-3′	786-805
*virK*	SUP10250	Sense	5′-AACCACTGTTTACCCTCGCG-3′	434-453
		Antisense	5′-AGGTGGCGAATCATCTCCTG-3′	532-551
*mdh*	SUP14945	Sense	5′-TGCTGTCACAAGTGGAAGGT-3′	554-573
		Antisense	5′-CGCTTTGGCTTCAACCACTT-3′	635-654

* *V. mimicus* strain NCTC11435 (GenBank: GCA_900460385/NZ_UHIG00000000).

**Table 2:** MIC and resistance category for *V. mimicus* strain CS-66.

Antibiotic	MIC * (μg/mL)	Category	Sub-MIC (μg/mL)
Polymyxin B (PL-B)	6.25	Resistant	2.0
Colistin (CL)	6.25	Resistant	2.0
Kanamycin (KM)	25.0	Intermediate	8.0
Chloramphenicol (CP)	1.56	Susceptible	0.5
Ciprofloxacin (CPFX)	0.00625	Susceptible	0.002

* Minimum inhibitory concentration.

**Table 3:** Growth of *V. mimicus* strain CS-66 in LB broth containing a sub-MIC of antibiotic.

Antibiotic	Lag Phase	Cultivation Period to Reach	
		Mid-Log Phase	Late-Log Phase
None	<1 h	3 h	9 h
Polymyxin B (PL-B)	9 h	11–13 h	17–19 h
Colistin (CL)	9 h	11–13 h	17–19 h
Kanamycin (KM)	9 h	13–15 h	>20 h
Chloramphenicol (CP)	1 h	7–9 h	>20 h
Ciprofloxacin (CPFX)	1 h	9–11 h	>20 h

## Data Availability

The original contributions presented in this study are included in the article. Further inquiries can be directed to the corresponding author.

## References

[1] Schulz G.E. (2002). The structure of bacterial outer membrane proteins. Biochim. Biophys. Acta.

[2] Nikaido H. (2003). Molecular basis of bacterial outer membrane permeability revisited. Microbiol. Mol. Biol. Rev..

[3] Pohlner J., Meyer T.F., Jalajakumari M.B., Manning P.A. (1986). Nucleotide sequence of *ompV*, the gene for a major *Vibrio cholerae* outer membrane protein. Mol. Gen. Genet..

[4] Kaur D., Mukhopadhaya A. (2020). Outer membrane protein OmpV mediates *Salmonella enterica* serovar Typhimurium adhesion to intestinal epithelial cells via fibronectin and α_1_β_1_ integrin. Cell. Microbiol..

[5] Wu L., Lin X., Wang F., Ye D., Xiao X., Wang S., Peng X. (2006). OmpW and OmpV are required for NaCl regulation in *Photobacterium damselae*. J. Proteome Res..

[6] Xu C., Ren H., Wang S., Peng X. (2004). Proteomic analysis of salt-sensitive outer membrane proteins of *Vibrio parahaemolyticus*. Res. Microbiol..

[7] Xu C., Wang S., Ren H., Lin X., Wu L., Peng X. (2005). Proteomic analysis on the expression of outer membrane proteins of *Vibrio alginolyticus* at different sodium concentrations. Proteomics.

[8] Den-Feng Z., Hui L., Xiang-Min L., Xuan-Xian P. (2015). Outer membrane proteomics of kanamycin-resistant *Escherichia coli* identified MipA as a novel antibiotic resistance-related protein. FEMS Microbiol. Lett..

[9] Kaur D., Gandhi S., Mukhopadhaya A. (2021). *Salmonella* Typhimurium adhesin OmpV activates host immunity to confer protection against systemic and gastrointestinal infection in mice. Infect. Immun..

[10] Miyoshi S., Kumagai M., Tnanida R., Soda K., Yoshimoto Y., Mizuno T. (2022). Inhibitory effects of polymyxin B and human LL-37 on the flagellin expression in *Vibrio vulnificus*. Biocontrol Sci..

[11] Janda J.M., Powers C., Bryant R.G., Abbott S. (1988). Current perspectives on the epidemiology and pathogenesis of clinically significant *Vibrio* spp. Clin. Microbiol. Rev..

[12] Mohapatra S.S., Dwibedy S.K., Padhy I. (2021). Polymyxins, the last-resort antibiotics: Mode of action, resistance emergence, and potential solutions. J. Biosci..

[13] Davis B.R., Fanning G.R., Madden J.M., Steigerwalt A.G., Bradford H.B., Smith H.L., Brenner D.J. (1981). Characterization of biochemically atypical *Vibrio cholerae* strains and designation of a new pathogenic species, *Vibrio mimicus*. J. Clin. Microbiol..

[14] Herrera C.M., Crofts A.A., Henderson J.C., Pingali S.C., Davies B.W., Trent M.S. (2014). The *Vibrio cholerae* VprA-VprB two-component system controls virulence through endotoxin modification. mBio.

[15] Bilecen K., Fong J.C., Cheng A., Jones C.J., Zamorano-Sanchez D., Yildiz F.H. (2015). Polymyxin B resistance and biofilm formation in *Vibrio cholerae* are controlled by the response regulator CarR. Infect. Immun..

[16] The Japan Society of Chemotherapy (1990). Method for determination of minimum inhibitory concentration (MIC) by microdilution: The standard method of Japan Society of Chemotherapy. Chemotherapy.

[17] (2024). Performance Standards for Antimicrobial Susceptibility Testing: 34th Edition.

[18] Miyoshi S., Amako K., Muraoka M., Morinaga H., Ueba S. (2024). Mobile genetic elements associated with utilization of dichloromethane and methanol as energy sources in *Cupriavidus metallidurans*. J. Microorg. Control.

[19] Abramson J., Adler J., Dunger J., Evans R., Green T., Pritzel A., Ronneberger O., Willmore L., Ballad A.J., Bambrick J. (2024). Accurate structure prediction of biomolecular interactions with AlphaFold 3. Nature.

[20] Meng E.C., Goddard T.D., Pettersen E.F., Couch G.S., Pearson Z.J., Morris J.H., Ferrin T.E. (2023). UCSF ChimeraX: Tools for structure building and analysis. Protein Sci..

[21] Jurrus E., Engel D., Star K., Monson K., Brandi J., Felberg L.E., Brooks D.H., Wilson L., Chen J., Liles K. (2018). Improvements to the APBS biomolecular solvation software suite. Protein Sci..

[22] Ye B., Tian W., Wang B., Liang J. (2024). CASTpFold: Computed atlas of surface topography of the universe of protein folds. Nucleic Acids Res..

[23] Ko D., Sung D., Kim T.Y., Choi G., Bang Y.J., Choi S.H. (2023). CarRS two-component system essential for polymyxin B resistance of *Vibrio vulnificus* responds to multiple host environmental signals. Microbiol. Spectr..

[24] Mathieu-Denoncourt A., Whitfield G.B., Vincent A.T., Berne C., Pauzé-Foixet J., Mahieddine F.C., Brun Y.V., Duperthuy M. (2025). The *carRS-ompV-virK* operon of *Vibrio cholerae* senses antimicrobial peptides and activates the expression of multiple resistance systems. Sci. Rep..

[25] National Center for Biotechnology Information (NCBI). https://www.ncbi.nlm.nih.gov.

[26] Alhede M., Kragh K.N., Qvortrup K., Allesen-Holm M., Gennip M., Christensen L.D., Jensen P.Ø., Nielsen A., Parsek M., Wozniak D. (2011). Phenotypes of non-attached *Pseudomonas aeruginosa* aggregates resemble surface attached biofilm. PLoS ONE.

[27] Haaber J., Cohn M.T., Frees D., Andersen T.J., Ingmer H. (2012). Planktonic aggregates of *Staphylococcus aureus* protect against common antibiotics. PLoS ONE.

